# Comparative Performance of Private Equity–Owned US Nursing Homes During the COVID-19 Pandemic

**DOI:** 10.1001/jamanetworkopen.2020.26702

**Published:** 2020-10-28

**Authors:** Robert Tyler Braun, Hyunkyung Yun, Lawrence P. Casalino, Zachary Myslinski, Farai M. Kuwonza, Hye-Young Jung, Mark Aaron Unruh

**Affiliations:** 1 Department of Population Health Sciences, Weill Cornell Medical College, New York, New York

## Abstract

**Importance:**

It is not known whether nursing homes with private equity (PE) ownership have performed better or worse than other nursing homes during the coronavirus disease 2019 (COVID-19) pandemic.

**Objective:**

To evaluate the comparative performance of PE-owned nursing homes on COVID-19 outcomes.

**Design, Setting, and Participants:**

This cross-sectional study of 11 470 US nursing homes used the Nursing Home COVID-19 Public File from May 17, 2020, to July 2, 2020, to compare outcomes of PE-owned nursing homes with for-profit, nonprofit, and government-owned homes, adjusting for facility characteristics.

**Exposure:**

Nursing home ownership status.

**Main Outcomes and Measures:**

Self-reported number of COVID-19 cases and deaths and deaths by any cause per 1000 residents; possessing 1-week supplies of personal protective equipment (PPE); staffing shortages.

**Results:**

Of 11 470 nursing homes, 7793 (67.9%) were for-profit; 2523 (22.0%), nonprofit; 511 (5.3%), government-owned; and 543 (4.7%), PE-owned; with mean (SD) COVID-19 cases per 1000 residents of 88.3 [2.1], 67.0 [3.8], 39.8 [7.6] and 110.8 [8.1], respectively. Mean (SD) COVID-19 deaths per 1000 residents were 61.9 [1.6], 66.4 [3.0], 56.2 [7.3], and 78.9 [5.9], respectively; mean deaths by any cause per 1000 residents were 78.1 [1.3], 91.5 [2.2], 67.6 [4.5], and 87.9 [4.8], respectively. In adjusted analyses, government-owned homes had 35.5 (95% CI, −69.2 to −1.8; *P* = .03) fewer COVID-19 cases per 1000 residents than PE-owned nursing homes. Cases in PE-owned nursing homes were not statistically different compared with for-profit and nonprofit facilities; nor were there statistically significant differences in COVID-19 deaths or deaths by any cause between PE-owned nursing homes and for-profit, nonprofit, and government-owned facilities. For-profit, nonprofit, and government-owned nursing homes were 10.5% (9.1 percentage points; 95% CI, 1.8 to 16.3 percentage points; *P* = .006), 15.0% (13.0 percentage points; 95% CI, 5.5 to 20.6 percentage points; *P* < .001), and 17.0% (14.8 percentage points; 95% CI, 6.5 to 23.0 percentage points; *P* < .001), respectively, more likely to have at least a 1-week supply of N95 masks than PE-owned nursing homes. They were 24.3% (21.3 percentage points; 95% CI, 11.8 to 30.8 percentage points; *P* < .001), 30.7% (27.0 percentage points; 95% CI, 17.7 to 36.2 percentage points; *P* < .001), and 29.2% (25.7 percentage points; 95% CI, 16.1 to 35.3 percentage points; *P* < .001) more likely to have a 1-week supply of medical gowns than PE-owned nursing homes. Government nursing homes were more likely to have a shortage of nurses (6.9 percentage points; 95% CI, 0.0 to 13.9 percentage points; *P* = .049) than PE-owned nursing homes.

**Conclusions and Relevance:**

In this cross-sectional study, PE-owned nursing homes performed comparably on staffing levels, resident cases, and deaths with nursing homes with other types of ownership, although their shortages of PPE may warrant monitoring.

## Key Points

**Question:**

Compared with other nursing homes, are private equity (PE)–owned nursing homes associated with better or worse coronavirus disease 2019 (COVID-19) outcomes?

**Findings:**

In this cross-sectional study of 11 470 US nursing homes, there were no statistically significant differences in staffing levels, COVID-19 cases or deaths, or deaths from any cause between PE nursing homes and facilities with other ownership types. Compared with PE, all other ownership types were more likely to have at least a 1-week supply of N95 masks and medical gowns.

**Meaning:**

In this study, PE-owned nursing homes performed comparably with for-profit and nonprofit nursing homes based on COVID-19 cases and deaths and deaths by any cause but had less personal protective equipment than other nursing homes.

## Teaser

This cross-sectional study evaluates the comparative performance of private equity–owned nursing homes on coronavirus disease 2019 (COVID-19) outcomes.

## Introduction

Nursing homes have been disproportionately affected by the coronavirus disease 2019 (COVID-19) pandemic. These facilities provide care to some of the nation’s most at-risk patients, including individuals receiving postacute care and long-stay residents requiring 24-hour custodial care, most of whom are older adults. Although there are only 1.3 million patients in US nursing homes,^1^ representing less than 0.4% of the country’s population,^2^ 43% of all COVID-19 deaths have been attributed to these facilities.^3^

For more than 2 decades, private equity (PE) firms have been acquiring nursing homes.^4^ This trend has raised concerns among policy makers regarding the quality of care provided by facilities owned by these firms.^5^ The disproportionate impact of the COVID-19 pandemic on nursing homes has amplified these concerns.^6^

PE firms typically make acquisitions with the expectation of high short-term returns on investment of 20% or more annually.^7^ Opponents of PE ownership of nursing homes suggest that these firms will prioritize profit over patient care because of pressure to increase the returns of investors.^8^ Unlike for-profit nursing homes that may have longer-term business plans, PE ownership may have little experience in nursing home care and may focus on selling acquired facilities within a short time, typically 3 to 5 years.^9^ Furthermore, PE firms often use leveraged buyouts for acquisitions, and the nursing homes are responsible for payments on the loans that PE firms use to acquire them.^10^ The targeting of short time frames before selling nursing homes for large returns on investment combined with large amounts of debt might lead PE-owned nursing homes to implement cost-cutting practices, such as reducing staffing levels. Staffing is the largest expenditure for nursing homes, accounting for approximately half the cost of providing care,^11^ making reduced staffing levels an attractive strategy to increase profits. Lower staffing levels in nursing homes have been associated with poorer quality care.^12,13,14^ PE-owned nursing homes might also attempt to attract more profitable patients, such as postacute patients covered by Medicare, which has high reimbursements compared with those for Medicaid patients, ie, for custodial care.^15^

Proponents of PE argue that these firms can bring management expertise that improves the quality and efficiency of nursing home care, eg, through better workforce management. Similarly, PE firms can bring capital to improve nursing homes’ health information technology (IT) infrastructure, an area in which they have lagged behind other health care professionals, facilities, and systems.^16^ Patients in nursing homes are often exposed to fragmented and poorly coordinated care,^17,18^ which can potentially be improved by increasing health IT capabilities. Moreover, proponents note that the nursing home industry has a history of poor regulatory compliance^19^ and that PE firms can use their management expertise, health IT, and legal resources to improve compliance.

Prior studies examining the association of PE ownership with nursing home quality and staffing have had mixed results.^3,9,15,20,21,22,23^ To our knowledge, no study to date has analyzed the comparative performance of PE-owned nursing homes during the COVID-19 pandemic. We used a nationwide sample to compare COVID-19 cases and deaths among patients in PE nursing homes with those among patients in nonprofit, government-owned, and (non-PE) for-profit nursing homes. We also evaluated the likelihood of having personal protection equipment (PPE) and staffing shortages based on ownership.

## Methods

The study was determined to be exempt by the institutional review board of Weill Cornell Medical College because it did not involve human participants. Therefore, informed consent was not required. This study followed the Strengthening the Reporting of Observational Studies in Epidemiology (STROBE) reporting guideline.

### Study Data and Participants

We identified nursing home acquisitions by PE firms between 2010 and 2020 using the S&P Capital IQ, Irving Levin Associates Health Care M&A, and Centers for Medicare & Medicaid Services (CMS) Nursing Home Compare Ownership databases. The S&P and Irving Levin databases report acquisition details that include acquisition announcement date, name of the acquired nursing home, the platform nursing home that acquired the nursing home, and the PE firm that owns the nursing home. The Nursing Home Compare database provides a CMS Certification Number (CCN), facility name, facility address, owner name, and the date on which ownership began.

Acquisitions by PE firms were identified in 2 ways. First, we confirmed PE acquisitions in the S&P and Irving Levin databases by manually reviewing each acquirers’ company profile using CB Insights, Bloomberg Businessweek, Pitchbook, and web-based searches to see whether they were a PE firm or PE-backed platform nursing home (eAppendix in the Supplement). Second, we used keyword searches in the Nursing Home Compare database to identify PE firms and PE-backed platform nursing homes that were not in the S&P and Irving Levin databases. The identified acquisitions were then manually matched to CMS Provider of Services data using nursing home name, address, and location to obtain the CCN. More details on the identification of PE acquisitions, in addition to the geographic distribution of acquisitions, are provided in the eAppendix and eTable 1 in the Supplement, respectively.

Using nursing home CCNs, we merged 2 additional data sources. First, we merged the acquisition database with the 2017 Long-term Care: Facts on Care in the US (LTCFocus) database to obtain nursing home patient and facility characteristics. Second, we merged the CMS Nursing Home COVID-19 Public File as of July 2, 2020, to obtain COVID-19–related measures. Nursing homes were required to begin reporting cumulative cases and deaths for the database beginning May 17, 2020, with weekly updates thereafter. We excluded hospital-based nursing homes, nursing homes with incomplete patient or facility information, and those that did not pass the US Centers for Disease Control and Prevention (CDC) quality assurance check.^24^ Data quality assurance checks were performed by CMS on 8 data fields. If values in the data fields were implausible (eg, the number of deaths was implausibly high compared with the number of beds in the facility), they were flagged as not passing the quality assurance check. Unadjusted COVID-19 outcomes for nursing homes that did not pass the CDC quality assurance check appear in eTable 2 in the Supplement. The final sample included 11 470 nursing homes (eFigure in the Supplement).

### Study Variables

#### Outcome Measures

From the CMS Nursing Home COVID-19 Public File, our outcomes included self-reported measures of COVID-19 cases and deaths and deaths by any cause; 5 measures of nursing home PPE supplies; and 4 measures of nursing home staffing. We measured total resident COVID-19 confirmed cases, total resident COVID-19 deaths, and deaths by any cause per 1000 residents. Total resident COVID-19 deaths were defined as residents with suspected or laboratory-positive COVID-19 tests who died in the nursing home or another location. Nursing homes that had a COVID-19 death must have reported at least 1 COVID-19 confirmed case. For PPE supplies, we created separate dichotomous measures for whether a nursing home reported at least a 1-week supply of N95 masks, eye protection, medical gowns, gloves, and hand sanitizer. For staffing, we created dichotomous measures for whether a nursing home reported having shortages of nursing, clinical, aid, or other personnel.

#### Covariates

From LTCFocus, the covariates used in our adjusted analyses included nursing home characteristics (ie, mean resident age, percentage women, occupancy rate, mean activities of daily living score, multifacility chain membership), including terciles of the distributions of the percentage of patients covered by Medicaid, percentage covered by Medicare, percentage of White residents, and the total number of beds. An indicator of rural location was derived from the US Department of Agriculture Rural-Urban Commuting Areas database.^25^

### Statistical Analysis

We conducted 3 analyses. First, we made unadjusted comparisons of facility characteristics by category of ownership using 1-way analysis of variance for continuous measures and χ^2^ tests for categorical measures (Table 1). Second, we made unadjusted comparisons for each outcome measure among other nursing home ownership categories compared with PE-owned nursing homes using *t* tests for continuous measures and proportional tests for dichotomous measures (Table 2). Third, we conducted the same comparisons while adjusting for the covariates described earlier using linear regression models. In all analyses, comparisons were made of nursing homes in the same Hospital Referral Region (HRRs) by including HRR fixed effects. Relative differences were derived by dividing estimates for continuous measures by the unadjusted mean of the outcome across all ownership types and by dividing estimates for dichotomous measures by the unadjusted proportions across all ownership types (eTable 3 in the Supplement). Standard errors were adjusted for clustering at the HRR level, and Bonferroni correction was used to adjust for multiple comparisons. All continuous outcome measures were winsorized at the top 1% of the distribution to exclude outliers. In sensitivity analyses, we addressed the skewness of our data by using Poisson regressions for continuous outcome measures and logistic regressions for dichotomous measures. Statistical analysis was conducted in Stata version 16.0 (StataCorp). Statistical significance was set at *P* < .05, and all tests were 2-tailed.

**Table 1.** Characteristics of Nursing Homes by Ownership

Variable^a^	Nursing homes, No. (%)				*P* value^b^
	For-profit, excluding private equity (n = 7793)	Private equity–owned (n = 543)	Nonprofit (n = 2523)	Government-owned (n = 611)	
Nursing home characteristics					
Total beds, tercile					
Lowest	2260 (29.0)	161 (29.7)	1169 (46.3)	233 (38.1)	<.001
Middle	3095 (39.7)	228 (42.0)	722 (28.6)	162 (26.5)	
Highest	2438 (31.3)	154 (28.4)	632 (25.1)	216 (35.4)	
Occupancy rate, mean (SD), %	72.6 (15.3)	74.6 (15.1)	75.7 (14.3)	72.3 (15.7)	<.001
ADL score, mean (SD)^c^	16.6 (2.7)	17.1 (1.8)	17.0 (2.3)	16.0 (2.6)	<.001
Multifacility chain membership	4855 (62.3)	497 (91.5)	1190 (47.2)	214 (35.0)	<.001
Rural	390 (5.0)	26 (4.8)	255 (10.1)	110 (18.0)	<.001
Patient characteristics					
Patient age, mean (SD), y	78.1 (6.4)	78.1 (5.3)	83.9 (6.7)	80.1 (5.9)	<.001
Women residents, mean (SD), %	64.8 (11.6)	65.2 (10.0)	71.9 (10.9)	64.0 (16.9)	<.001
Percentage White residents, tercile					
Lowest	3131 (40.2)	197 (36.3)	397 (15.7)	139 (22.8)	<.001
Middle	2697 (34.6)	203 (37.4)	731 (29.0)	182 (29.8)	
Highest	1965 (25.2)	143 (26.3)	1395 (55.3)	290 (47.5)	
Percentage Medicare residents, tercile					
Lowest	2510 (32.2)	118 (21.7)	857 (34.0)	298 (48.8)	<.001
Middle	2621 (33.6)	202 (37.2)	823 (32.6)	208 (34.0)	
Highest	2662 (34.2)	223 (41.1)	843 (33.4)	105 (17.2)	
Percentage Medicaid residents, tercile					
Lowest	2087 (26.8)	134 (24.7)	1480 (58.7)	181 (29.6)	<.001
Middle	2736 (35.1)	198 (36.5)	657 (26.0)	207 (33.9)	
Highest	2970 (38.1)	211 (38.9)	386 (15.3)	223 (36.5)	

Abbreviation: ADL, activities of daily living.

^a^ Facility and patient characteristics data are from the 2017 Long-term Care: Facts on Care in the US and the US Department of Agriculture Rural-Urban Commuting Areas database.

^b^ Unadjusted comparisons of facility and patient characteristics by ownership category were made using 1-way analysis of variance for continuous measures and χ^2^ tests for categorical measures.

^c^ The ADL score ranges from 0 to 28, based on a score of 0 to 4 on 7 different ADLs, with 0 indicating completely independent and 28, completely dependent.

**Table 2.** Unadjusted COVID-19 Outcomes by Ownership

Outcome^a^	% (SD)		*P* value^b^	Nonprofit, % (SD), (n = 2523)	*P* value^b^	Government, % (SD), (n = 611)	*P* value^b^
	Private equity–owned (n = 543)	For-profit, excluding private equity (n = 7793)					
Resident COVID-19 measures, mean (SD), per 1000 residents							
Confirmed COVID-19 cases	110.8 (8.1)	88.3 (2.1)	.007	67.0 (3.8)	<.001	39.8 (7.6)	<.001
COVID-19 deaths	78.9 (5.9)	61.9 (1.6)	.006	66.4 (3.0)	.06	56.2 (7.3)	.02
All deaths	87.9 (4.8)	78.1 (1.3)	.047	91.5 (2.2)	.50	67.6 (4.5)	.002
PPE supply measures							
1-wk supply of N95 masks	76.8 (1.5)	86.2 (0.4)	<.001	89.1 (0.7)	<.001	91.8 (1.4)	<.001
1-wk supply of surgical masks	94.1 (1.0)	93.0 (0.3)	.30	95.5 (0.5)	.23	96.2 (1.0)	.14
1-wk supply of eye protection	93.2 (1.0)	93.4 (0.3)	.87	95.8 (0.5)	.02	94.9 (1.0)	.22
1-wk supply of medical gowns	64.3 (1.4)	88.0 (0.4)	<.001	91.4 (0.6)	<.001	91.7 (1.3)	<.001
1-wk supply of gloves	94.3 (0.8)	95.7 (0.2)	.10	97.4 (0.3)	.001	97.9 (0.7)	.002
1-wk supply of hand sanitizer	93.9 (0.9)	94.8 (0.2)	.38	96.5 (0.4)	.01	96.7 (0.9)	.03
Staff shortage measures							
Shortage of nursing staff	10.9 (1.5)	15.5 (0.4)	.003	13.6 (0.7)	.11	20.3 (1.4)	<.001
Shortage of clinical staff	2.0 (0.7)	2.9 (0.1)	.25	2.6 (0.3)	.45	3.0 (0.6)	.34
Shortage of aides	13.3 (1.6)	18.1 (0.4)	.004	16.0 (0.8)	.13	21.6 (1.5)	<.001
Shortage of other staff	7.4 (1.2)	9.0 (0.3)	.20	8.5 (0.6)	.39	11.5 (1.2)	.02

Abbreviations: COVID-19, coronavirus disease 2019; PPE, personal protective equipment.

^a^ Outcome measures are from the Centers for Medicare & Medicaid Services Nursing Home COVID-19 Public File as of July 2, 2020.

^b^ Unadjusted comparisons of outcome measures by ownership category relative to private equity were made using *t* tests for resident COVID-19 measures and proportional tests for resident PPE supply and staff shortage measures.

## Results

### Nursing Home Characteristics by Ownership

Table 1 presents unadjusted nursing home and patient characteristics by 4 categories of ownership: for-profit (non-PE), nonprofit, government-owned, and PE-owned. Of the 11 470 nursing homes in our sample, 7793 (67.9%) were for-profit, 2523 (22.0%) were nonprofit, 611 (5.3%) were government-owned, and 543 (4.7%) were PE-owned. Nursing home characteristics were generally similar across ownership categories. However, 497 PE-owned nursing homes (91.5%) were part of multifacility chains compared with 4855 for-profit nursing homes (62.3%), 1190 nonprofit nursing homes (47.2%), and 214 government-owned nursing homes (35.0%). Patient characteristics were similar across ownership types, although more PE-owned nursing homes were in the highest tercile of percentage of patients with Medicare: 223 PE-owned nursing homes (41.1%) vs 2662 for-profit nursing homes (34.2%), 843 nonprofit nursing homes (33.4%), and 105 government-owned nursing homes (17.2%).

### Unadjusted Differences in Outcomes by Ownership

In unadjusted analyses (Table 2) for mean (SD) number of confirmed COVID-19 cases per 1000 residents, PE-owned nursing homes had the highest number (110.8 [8.1]) compared with for-profit (88.3 [2.1]; *P* = .007), nonprofit (67.0 [3.8]; *P* < .001), and government-owned (39.8 [7.6]; *P* < .001). For mean (SD) number of confirmed COVID-19 deaths per 1000 residents, PE-owned nursing homes also had the highest number (78.9 [5.9]) compared with for-profit (61.9 [1.6]; *P* = .006), nonprofit (66.4 [3.0]; *P* = .06), and government-owned (56.2 [7.3]; *P* = .02) nursing homes. Nonprofit nursing homes had the highest mean (SD) number of deaths by any cause per 1000 residents (eg, PE vs nonprofit: 87.9 [4.8] vs 91.5 [2.2]; *P* = .50). Relative to PE nursing homes, for-profit nursing homes (78.1 [1.3]; *P* = .047) and government-owned nursing homes (67.6 [4.5]; *P* = .002) had fewer deaths from any cause per 1000 residents.

For N95 masks, PE-owned nursing homes reported having the lowest mean (SD) percentage of facilities with a 1-week supply of N95 masks (76.8% [1.5%]) and medical gowns (64.3% [1.4%]) compared with government-owned (masks: 91.8% [1.4%]; *P* < .001; gowns: 91.7% [1.3%]; *P* < .001), nonprofit (masks: 89.1% [0.7%]; *P* < .001; gowns: 91.4% (0.6%); *P* < .001), and for-profit nursing homes (masks: 86.2% (0.4%); *P* < .001; gowns: 88.0% [0.4%]; *P* < .001). Compared with PE-owned nursing homes, a higher percentage of nonprofit nursing homes reported having a 1-week supply of eye protection (93.2% [1.0%] vs 95.8% [0.5%]; *P* = .02), gloves (94.3% [0.8%] vs 97.4% [0.3%]; *P* < .001), and hand sanitizer (93.9% [0.9%] vs 96.5% [0.4%]; *P* = .01). Government-owned nursing homes were also more likely to have a 1-week supply of medical gloves (97.9% [0.7%]; *P* = .002) and hand sanitizer (96.7% [0.9%]; *P* = .03) compared with PE-owned nursing homes.

PE-owned nursing homes reported having the lowest percentage with a shortage of nurses (10.9% [1.5%]), clinical staff (2.0% [0.7%]), aides (13.3% [1.6%]), and other staff (7.4% [1.2%]) compared with for-profit (nursing staff: 15.5% [0.4%]; *P* < .003; clinical staff: 2.9% [0.1%]; *P* = .11; aids: 18.1% [0.4%]; *P* < .004; other staff: 9.0% [0.3%]; *P* = .20), government-owned (nursing staff: 20.3% [1.4%]; *P* < .001; clinical staff: 3.0% [0.6%]; *P* = .34; aids: 21.6% [1.5%]; *P* < .001; other staff: 11.5% [1.2%]; *P* = .02), and nonprofit (nursing staff: 13.6% [0.7%]; *P* = .25; clinical staff: 2.6% (0.3%); *P* = .45; aids: 16.0% [0.8%]; other staff: 8.5% [0.6%]; *P* = .39) nursing homes.

### Adjusted Differences in Outcomes by Ownership

In multivariate analyses (Table 3), government-owned nursing homes reported 35.5 (95% CI, −69.2 to −1.8; *P* = .03) fewer COVID-19 confirmed cases per 1000 residents compared with PE nursing homes (eTable 4, eTable 5, and eTable 6 in the Supplement). However, COVID-19 deaths and deaths by any cause per 1000 residents did not differ significantly between PE-owned and nonprofit or between PE-owned vs government-owned nursing homes. COVID-19 confirmed cases, COVID-19 deaths, and deaths by any cause per 1000 residents did not differ significantly between PE-owned and for-profit nursing homes.

**Table 3.** Association Between COVID-19 Outcomes and Ownership

Outcome	For-profit, % (95% CI)^a^	*P* value^b^	Nonprofit, % (95% CI)^a^	*P* value^b^	Government-owned, % (95% CI)^a^	*P* value^b^
Resident COVID-19 measures, No. (95% CI), per 1000 residents						
Confirmed COVID-19 cases	−18.2 (−49.4 to 13.0)	.73	−25.6 (−57.4 to 6.2)	.20	−35.5 (−69.2 to −1.8)	.03
COVID-19 deaths	−5.3 (−27.4 to 16.9)	>.99	−8.9 (−32.0 to 14.1)	>.99	−6.7 (−35.0 to 21.7)	>.99
All deaths	9.0 (−22.4 to 4.3)	.44	−4.6 (−18.4 to 9.2)	>.99	−8.9 (−25.1 to 7.4)	.89
PPE supply measures						
1-wk supply of N95 masks	9.1 (1.8 to 16.3)	.006	13.0 (5.5 to 20.6)	<.001	14.8 (6.5 to 23.0)	<.001
1-wk supply of surgical masks	1.6 (−6.2 to 3.1)	>.99	2.5 (−2.3 to 7.3)	>.99	2.4 (−3.0 to 7.8)	>.99
1-wk supply of eye protection	−0.1 (−4.7 to 4.5)	>.99	3.7 (−1.2 to 8.6)	.29	2.3 (−3.2 to 7.8)	>.99
1-wk supply of gowns	21.3 (11.8 to 30.8)	<.001	27.0 (17.7 to 36.2)	<.001	25.7 (16.1 to 35.3)	<.001
1-wk supply of gloves	1.6 (−2.5 to 5.7)	>.99	3.0 (−1.3 to 7.2)	.39	3.3 (−1.0 to 7.7)	.25
1-wk supply of hand sanitizer	1.0 (−3.2 to 5.3)	>.99	2.7 (−1.8 to 7.3)	.65	1.8 (−3.4 to 6.9)	>.99
Staff shortage measures, %						
Shortage of nursing staff	3.2 (−1.4 to 7.7)	.41	1.0 (−4.0 to 6.0)	>.99	6.9 (0.0 to 13.9)	.049
Shortage of clinical staff	0.3 (−1.5 to 2.1)	>.99	0.0 (−2.0 to 2.1)	>.99	−0.2 (−3.0 to 2.7)	>.99
Shortage of aides	3.2 (−2.4 to 8.8)	.79	1.0 (−5.4 to 7.3)	>.99	4.7 (−2.9 to 12.2)	.60
Shortage of other staff	1.0 (−2.6 to 4.7)	>.99	0.3 (−3.6 to 4.1)	>.99	2.5 (−2.6 to 7.7)	>.99

Abbreviations: COVID-19, coronavirus disease 2019; PPE, personal protective equipment.

^a^ Linear regressions were used for estimation. All models included the following covariates: mean age of residents; percentage women; occupancy rate; mean activities of daily living score; multifacility chain membership; rural status; terciles of the distributions of the percentage of patients covered by Medicaid, percentage of patients covered by Medicare, and percentage of White patients; and total number of beds. Private equity ownership is the comparison group for all models. Standard errors were adjusted for clustering at the level of the Hospital Referral Region.

^b^ Bonferroni correction was used for multiple comparisons.

Adjusted estimates for PPE measures indicated that for-profit, nonprofit, and government nursing homes were 10.5% (9.1 percentage points; 95% CI, 1.8 to 16.3 percentage points; *P* = .006), 15.0% (13.0 percentage points; 95% CI, 5.5 to 20.6 percentage points; *P* < .001), and 17.0% (14.8 percentage points; 95% CI, 6.5 to 23.0 percentage points; *P* < .001), respectively, more likely to report having at least a 1-week supply of N95 masks compared with PE-owned nursing homes. Additionally, for-profit, nonprofit, and government-owned nursing homes were 24.3% (21.3 percentage points; 95% CI, 11.8 to 30.8 percentage points; *P* < .001), 30.7% (27.0 percentage points; 95% CI, 17.7 to 36.2 percentage points; *P* < .001), and 29.2% (25.7 percentage points; 95% CI, 16.1 to 35.3 percentage points; *P* < .001), respectively, more likely to have at least a 1-week supply of medical gowns than PE-owned nursing homes. All other associations between PE-owned and for-profit, nonprofit, and government-owned nursing homes for PPE outcomes were not statistically different (Table 3).

Staffing shortages did not significantly differ between PE-owned and for-profit nursing homes or between PE-owned and nonprofit nursing homes. However, government-owned nursing homes had a 6.9 percentage point (95% CI, 0.0 to 13.9 percentage points; *P* = .049) higher probability of having a shortage of nursing personnel, representing a relative difference of 45.8%.

Estimates from sensitivity analyses using Poisson regressions for continuous outcomes and logistic regressions for dichotomous outcomes were largely consistent with those from our primary analyses. eTable 7 and eTable 8 in the Supplement present these analyses.

## Discussion

In this national study of 11 470 nursing homes, PE-owned nursing homes did not have significantly higher self-reported rates of COVID-19 cases than nonprofit or for-profit nursing homes but had 35.5 more cases per 1000 residents than government-owned facilities. PE-owned homes did not have significantly higher rates of COVID-19 deaths or of deaths from any cause. It is possible that differences in rates of testing among facilities may have obscured differences in COVID-19 cases and deaths, but this would not have affected estimates of deaths by any cause.

By several measures, PE-owned facilities were less likely to have a 1-week or longer supply of PPE. PE-owned facilities were significantly less likely to have a 1-week supply of N95 masks and of gowns compared with all other types of facilities, less likely to have a supply of eye protection compared with nonprofit nursing homes, and less likely to have a supply of gloves compared with government-owned facilities. For example, PE-owned facilities were 14.8% less likely to have at least a 1-week supply of N95 masks compared with government facilities and 13.0% and 9.1% less likely to have them compared with nonprofit and for-profit nursing homes, respectively. There were no significant differences in staffing shortages between PE facilities and other types of facilities, except that government-owned facilities were 6.9% more likely to have a shortage of nursing staff.

It is not clear why PE-owned nursing homes had lower supplies of PPE. This may have been due to cost-cutting strategies undertaken by these facilities. If this was the case, it is not clear why staffing levels were not also lower for PE-owned nursing homes. It is possible that PE-owned homes were attempting to control costs by keeping the minimum level of supplies that they anticipated would be necessary.

Although no previous research that we are aware of has examined PE ownership and outcomes associated with COVID-19, 6 recent studies^4,26,27,28,29,30^ compared outcomes of for-profit nursing homes with nursing homes with other types of ownership. The findings of these studies were inconsistent. Two studies^4,26^ did not find an association between for-profit ownership and COVID-19 cases, 1 study^27^ found that for-profit ownership was associated with higher COVID-19 mortality rates, 2 studies^28,29^ did not find statistically significant associations between for-profit ownership and COVID-19 cases or deaths, and 1 study^30^ found both positive and negative associations of for-profit ownership with cases and deaths. These studies classified PE-owned nursing homes as for-profit entities and did not distinguish between the 2 types of ownership. Our study makes a new contribution by comparing PE with non-PE for-profit nursing homes in addition to other ownership categories, based on outcomes related to COVID-19. The distinction between PE and non-PE for-profit facilities is important because policy makers have expressed concerns that PE-owned nursing homes may have different incentives and provide lower quality care compared with for-profit as well as nonprofit homes.^5^

It is possible that nursing home characteristics, including past performance on quality measures, are not associated with whether COVID-19 makes its way into a facility, how rapidly it spreads once inside, or the related mortality rate after patients are exposed. For example, past scores on the CMS Five Star Rating system, used to measure nursing home quality, have not been associated with whether a facility has any COVID-19 cases or the number of cases among those with at least 1 case.^4^ Similarly, performance on the quality measures used to construct the Five Star Ratings has not been associated with the number of cases in nursing homes.^31^ Other factors, such as COVID-19 infection rates in the communities where staff reside^32^ and differences in state policies implemented to prevent the spread of the virus,^33^ may have stronger associations with COVID-19 morbidity and mortality in nursing homes.

Our study does not provide evidence regarding whether PE nursing homes perform better or worse than non-PE nursing homes on measures that more broadly reflect clinical quality. In studies conducted before the pandemic, 2 studies^9,23^ found little evidence of an association between PE ownership and nursing home performance on a variety of clinical process and outcome measures. Both studies were based on comparisons of nursing homes more than a decade ago. One of these studies,^9^ in addition to 3 others,^20,21,22^ examined PE ownership and nursing home staffing levels; 3 studies^9,20,21^ found little evidence of changes in staffing and 1 study^22^ identified declines in staffing. Two recent working papers^3,15^ also had mixed results. The first^15^ found PE ownership to be associated with declines in staffing levels, increased rehospitalization rates, and worse CMS Five Star Ratings. Conversely, the second working paper^3^ found that PE firms tended to increase staffing levels and improve the Five Star Ratings of nursing homes. The inconsistent results of the 2 studies may have been because of different lengths of study period or differences in their analytic approaches.

### Limitations

Our study has several limitations. First, results are based on cross-sectional data, and the estimates may not reflect causal relationships because we cannot exclude the possibility of unmeasured or unobserved differences between nursing homes. Second, PE acquisitions that were identified through the Capital IQ and Irving Levin databases were captured through public announcements, which likely underestimate the total number of acquisitions. Additionally, acquisitions that are not publicly announced, such as smaller acquisitions and those in which PE firms had minority ownership, may not be identified in the databases. This may bias our estimates toward no effect owing to measurement error. However, we found a similar number of nursing home acquisitions as 2 other studies.^3,15^ Third, 1232 nursing homes (8.6%) reported data that did not pass validity checks in the Nursing Home COVID-19 Public File. There may be differences between facilities that reported confirmed data and those that did not. Fourth, the Nursing Home COVID-19 Public File is being updated regularly as nursing homes report additional cases and deaths. Fifth, some nursing homes may not have tested for COVID-19 as much as others, which may have led to underreporting of COVID-19 cases and deaths by some facilities. Sixth, reporting requirements for COVID-19 deaths vary by state, and nursing home residents who were hospitalized prior to death may not have been counted toward facility totals in some cases. We compared nursing homes within geographic regions, but it is possible that the likelihood of hospitalization prior to death varied by type of ownership. Seventh, the data are self-reported; some nursing homes may have inaccurately reported PPE supplies and staffing levels. Despite these limitations, the Nursing Home COVID-19 Public File has a number of strengths for addressing this topic that are not found elsewhere, including a large national sample and detailed COVID-19 outcomes, which can be linked to other publicly available data sources to identify comprehensive information on nursing homes.

## Conclusions

In this cross-sectional study, PE-owned nursing homes did not have more COVID-19 confirmed cases than nonprofit or for-profit nursing homes nor did they differ significantly in COVID-19 deaths or deaths by any cause. They reported having less PPE than other facilities and similar staffing shortages. Further study, including longitudinal studies, are needed to determine whether PE-owned nursing homes perform better or worse than non–PE-owned nursing homes on broader measures of clinical quality and whether they are associated with higher or lower health care spending.

## Notes

Supplement. **eAppendix. **Identification of Private Equity Nursing Home Acquisitions **eTable 1. **Private Equity Acquisitions of Nursing Homes by State **eFigure. **Sample Selection **eTable 2. **Unadjusted COVID-19 Outcomes by Ownership for Nursing Homes That Did Not Pass CDC Quality Assurance Check **eTable 3. **Unadjusted Means and Proportions of COVID-19 Resident Outcome Measures **eTable 4. **Association Between COVID-19 Resident Outcomes and Nursing Home Characteristics **eTable 5. **Association Between PPE Supply Outcomes and Nursing Home Characteristics **eTable 6. **Association Between Staff Shortage Outcomes and Nursing Home Characteristics **eTable 7. **Poisson Regression Estimates: Association Between COVID-19 Resident Outcomes and Ownership **eTable 8. **Logistic Regression Estimates: Association Between PPE Supply/Staff Shortage Outcomes and Ownership
