# Supplementary material for: Comparative Performance of Private Equity–Owned US Nursing Homes During the COVID-19 Pandemic
Source: JAMA Netw Open. 2020 Oct 28;3(10):e2026702. doi: 10.1001/jamanetworkopen.2020.26702 (PMC7593807; doi:10.1001/jamanetworkopen.2020.26702)
Supplement: Supplement. — eAppendix. Identification of Private Equity Nursing Home Acquisitions eTable 1. Private Equity Acquisitions of Nursing Homes by State eFigure. Sample Selection eTable 2. Unadjusted COVID-19 Outcomes by Ownership for Nursing Homes That Did Not Pass CDC Quality Assurance Check eTable 3. Unadjusted Means and Proportions of COVID-19 Resident Outcome Measures eTable 4. Association Between COVID-19 Resident Outcomes and Nursing Home Characteristics eTable 5. Association Between PPE Supply Outcomes and Nursing Home Characteristics eTable 6. Association Between Staff Shortage Outcomes and Nursing Home Characteristics eTable 7. Poisson Regression Estimates: Association Between COVID-19 Resident Outcomes and Ownership eTable 8. Logistic Regression Estimates: Association Between PPE Supply/Staff Shortage Outcomes and Ownership [file jamanetwopen-e2026702-s001.pdf]

Supplemental Online Content

Braun RT, Yun H, Casalino LP, et al. Comparative performance of private equity–owned US nursing homes during the COVID-19 pandemic. *JAMA Netw Open*. 2020;3(10):e2026702. doi:10.1001/jamanetworkopen.2020.26702

eAppendix. Identification of Private Equity Nursing Home Acquisitions
eTable 1. Private Equity Acquisitions of Nursing Homes by State
eFigure. Sample Selection
eTable 2. Unadjusted COVID-19 Outcomes by Ownership for Nursing Homes That Did Not Pass CDC Quality Assurance Check
eTable 3. Unadjusted Means and Proportions of COVID-19 Resident Outcome Measures
eTable 4. Association Between COVID-19 Resident Outcomes and Nursing Home Characteristics
eTable 5. Association Between PPE Supply Outcomes and Nursing Home Characteristics
eTable 6. Association Between Staff Shortage Outcomes and Nursing Home Characteristics
eTable 7. Poisson Regression Estimates: Association Between COVID-19 Resident Outcomes and Ownership
eTable 8. Logistic Regression Estimates: Association Between PPE Supply/Staff Shortage Outcomes and Ownership

This supplemental material has been provided by the authors to give readers additional information about their work.

© 2020 Braun RT et al. *JAMA Network Open.*

eAppendix. Identification of Private Equity Nursing Home Acquisitions

Information on private equity (PE) acquisition of nursing homes from January 1, 2010 through February 5, 2020 was accessed from S&P Capital IQ and Irving Levin Associates Health Care M&A databases.[1] Acquisition data from Irving Levin Associates Health Care M&A included the name and a description of the acquired nursing home, city and state of the acquired facilities, name and business description of the acquirer, a description of the deal, and the announcement date of the deal. Acquisition data from S&P Capital IQ included the name and description of the acquired facility, name and business description of the acquirer, a description of the deal, and the announcement date of the deal.

Acquisitions from Irving Levin Associates and S&P Capital IQ databases were confirmed to be PE or a platform nursing home[2] by examining the acquirer's business description in both datasets. If the business description mentioned that the acquirer was a PE firm, raised capital to invest in companies that are not publicly traded, or a platform nursing home that was PE-backed, we considered this a PE acquisition. If we were unable to confirm a PE acquisition using the acquirer's business description in both datasets, we then examined the acquirer's business descriptions in CB Insights, Pitchbook, Bloomberg Businessweek, the acquirer's home website, and various web searches and news articles to confirm if they were a PE firm or platform nursing home.[3]

Confirmed acquisitions were then matched to their Centers for Medicare and Medicaid Services (CMS) Certification Number (CCN) by manually matching on facility name and geographic location from Provider of Services (POS) data across years 2010-2020. The CMS POS data contains all registered nursing homes in the United States and provides the facility name, address, and CCN. The CCN remains the same across years even if the ownership and/or name of the facility changes. For nursing home acquisitions that spanned multiple facilities in different geographic regions, we aggregated all facility locations in the POS files and matched them to all confirmed acquisitions.

After we confirmed PE acquisitions using S&P Capital IQ and Irving Levin Associates Health Care M&A databases, we used the CMS Nursing Home Compare ownership public use file to add additional PE acquisitions. This database provides ownership information for currently registered nursing homes in the United States. Facility information in this dataset included CCN, facility name, facility address, owner name,

[1] Nursing homes that were acquired prior to the study period (2000-2009) and were identified in the data were removed from the analysis (n=569).
[2] A platform nursing home is defined as the initial acquisition/investment that a PE firm uses to recruit more employees and acquire smaller nursing homes. After investment, the PE firm generally controls a majority of the economic and voting interest in the nursing home acquired by the platform nursing home, which allows it to restructure the facility's financial, governance, and operational arrangements.
[3] Some nursing homes were acquired multiple times by different PE firms. For these nursing homes, the date of the earliest transaction was considered to be that nursing home's PE acquisition date.

© 2020 Braun RT et al. *JAMA Network Open.*

and the date on which ownership began. The date that ownership began was considered the acquisition date. PE-acquired nursing homes were identified by (1) searching ownership data for known PE firms and PE-backed platform nursing homes from our confirmed PE acquisitions in the S&P Capital IQ and Irving Levin Associates Health Care M&A databases and (2) performing targeted keyword searches for "partners," "capital," and "investment" and manually reviewing nursing home owners with those keywords in their names. Acquisitions that were not in our confirmed acquisition data were added to complete the PE acquisition database.

© 2020 Braun RT et al. *JAMA Network Open.*

eTable 1. Private Equity Acquisitions of Nursing Homes by State

state	Number of private equity acquisitions in the PE database	Number of private equity acquisitions in the dataset	Number of private equity acquisitions in the sample
AL	12	0	0
AR	14	0	0
AZ	11	0	0
CA	171	52	30
CO	17	0	0
CT	21	0	0
DC	2	0	0
DE	8	0	0
FL	147	99	27
GA	15	13	10
HI	1	1	1
IA	13	10	4
ID	13	10	10
IL	40	30	6
IN	63	37	25
KS	49	29	15
KY	86	68	36
LA	3	3	2
MA	64	23	22
MD	43	30	27
ME	13	10	4
MI	52	33	5
MN	36	20	5
MO	31	22	2
MS	14	14	7
MT	6	3	3
NC	40	38	20
ND	3	2	0
NE	24	12	1
NH	23	20	9
NJ	58	38	33
NM	25	20	11
NV	10	5	1
NY	10	5	4
OH	128	78	30

© 2020 Braun RT et al. *JAMA Network Open.*

eTable 1. Private Equity Acquisitions of Nursing Homes by State			
state	Number of private equity acquisitions in the PE database	Number of private equity acquisitions in the dataset	Number of private equity acquisitions in the sample
OK	28	5	4
OR	1	0	0
PA	133	103	21
RI	11	8	8
SC	12	10	3
SD	20	16	2
TN	43	39	20
TX	116	62	39
UT	15	8	7
VA	38	33	20
VT	11	10	7
WA	24	20	18
WI	42	27	10
WV	52	45	33
WY	1	1	1
Total	1813	1112	543

© 2020 Braun RT et al. *JAMA Network Open.*

eFigure. Sample Selection

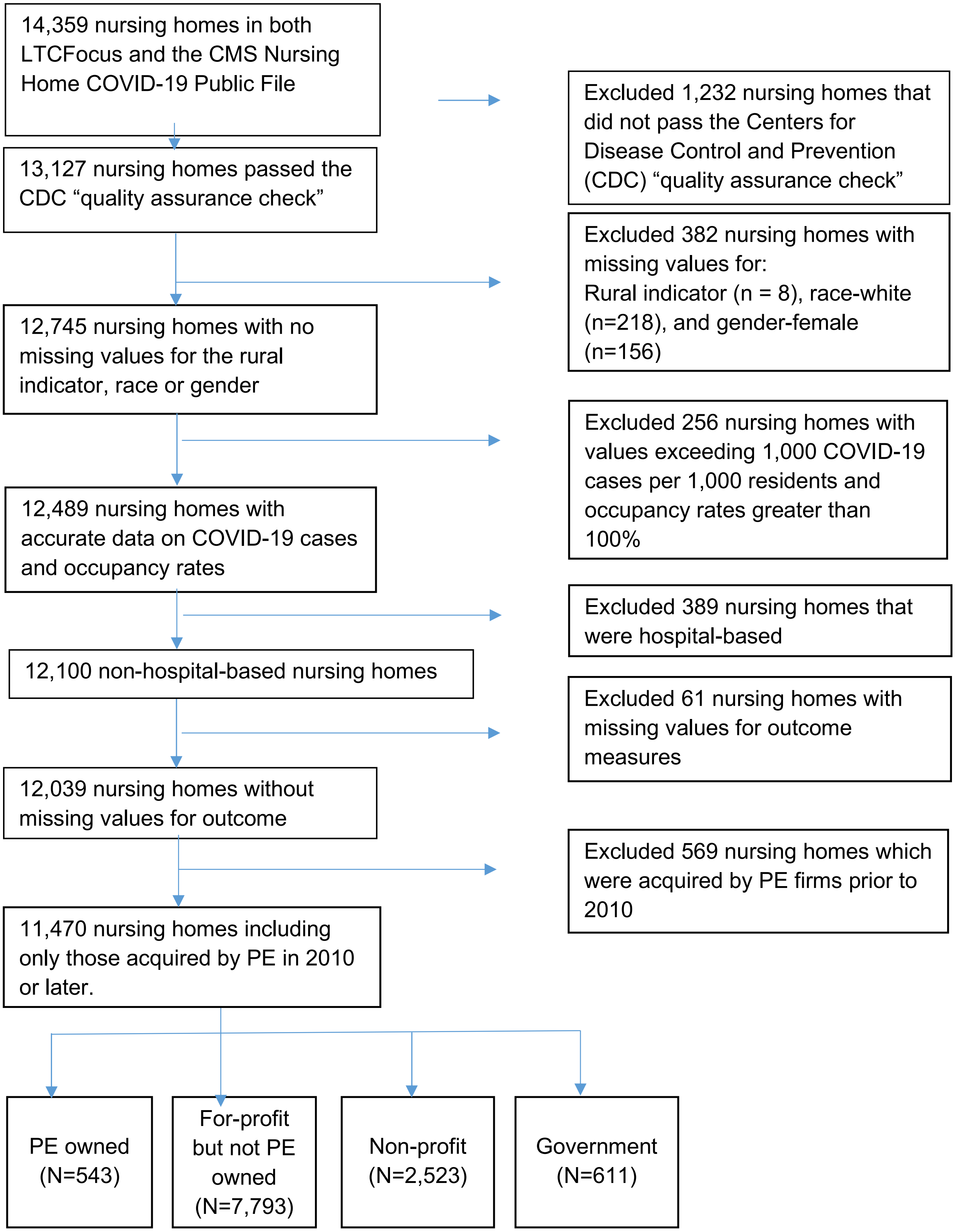

© 2020 Braun RT et al. *JAMA Network Open.*

eTable 2. Unadjusted COVID-19 Outcomes by Ownership for Nursing Homes That Did Not Pass CDC Quality Assurance Check

Outcome	For-profit, excluding private equity (N = 187)	Private equity (N = 9)	Non-profit (N = 50)	Government (N = 11)	P Value
Resident COVID-19 Measures					
COVID confirmed cases – per 1,000 residents	217.58	256.61	260.24	288.65	0.698
COVID deaths – per 1,000 residents	30.6	38.83	28.04	61.64	0.755
All deaths – per 1,000 residents	52.09	92.52	47.72	85.10	0.693
PPE Supply Measures					
One-week supply of N95 masks – no. (%)	171 (91.44)	8 (88.89)	50 (100.00)	9 (81.82)	0.093
One-week supply of surgical masks – no. (%)	179 (95.72)	9 (100.00)	48 (96.00)	10 (90.91)	0.793
One-week supply of eye protection – no. (%)	178 (95.19)	8 (88.89)	49 (98.00)	11 (100.00)	0.516
One-week supply of gowns – no. (%)	172 (91.98)	8 (88.89)	46 (92.00)	9 (81.82)	0.690
One-week supply of gloves – no. (%)	179 (95.72)	9 (100.00)	50 (100.00)	11 (100.00)	0.378
One-week supply of hand sanitizer – no. (%)	180 (96.26)	9 (100.00)	49 (98.00)	8 (72.73)	0.002
Staff Shortage Measures					
Shortage of nursing staff – no. (%)	40 (21.39)	0 (0.00)	10 (20.00)	2 (18.18)	0.481

© 2020 Braun RT et al. *JAMA Network Open.*

eTable 2. Unadjusted COVID-19 Outcomes by Ownership for Nursing Homes That Did Not Pass CDC Quality Assurance Check

Outcome	For-profit, excluding private equity (N = 187)	Private equity (N = 9)	Non-profit (N = 50)	Government (N = 11)	P Value
Shortage of clinical staff – no. (%)	5 (2.67)	0 (0.00)	4 (8.00)	0 (0.00)	0.251
Shortage of aides – no. (%)	47 (25.13)	1 (11.11)	11 (22.00)	1 (9.09)	0.496
Shortage of other staff – no. (%)	19 (10.16)	0 (0.00)	7 (14.00)	0 (0.00)	0.379

Abbreviations: PPE, personal protective equipment
We made unadjusted comparisons of outcome measures by ownership category using one-way analysis of variance (ANOVA) for Resident COVID-19 Measures and Chi-square tests for PPE supply and staff shortage measures.
Outcome measures are from the CMS Nursing Home COVID-19 Public File as of July 2, 2020.

© 2020 Braun RT et al. *JAMA Network Open.*

eTable 3. Unadjusted Means and Proportions of COVID-19 Resident Outcome Measures

**Outcome**	**Unadjusted Mean**
Resident COVID-19 Measures	
COVID confirmed cases per 1,000 residents	82.1
COVID deaths per 1,000 residents	63.5
All deaths per 1,000 residents	80.9
Personal Protection Equipment Supply Measures	
One-week supply of N95 masks (%)	86.7%
One-week supply of surgical masks (%)	93.8%
One-week supply of eye protection (%)	94.0%
One-week supply of gowns (%)	87.8%
One-week supply of gloves (%)	96.1%
One-week supply of hand sanitizer (%)	95.2%
Staff Shortage Measures	
Shortage of nursing staff (%)	15.1%
Shortage of clinical staff (%)	2.8%
Shortage of aides (%)	17.6%
Shortage of other staff (%)	9.0%
Abbreviations: PPE, personal protective equipment	

© 2020 Braun RT et al. *JAMA Network Open.*

eTable 4. Association Between COVID-19 Resident Outcomes and Nursing Home Characteristics

	COVID confirmed cases, per 1,000 residents		COVID deaths, per 1,000 residents		All deaths, per 1,000 residents	
**Predictor**	**Risk estimate (95% CI)**	**P Value**	**Risk estimate (95% CI)**	**P Value**	**Risk estimate (95% CI)**	**P Value**
Ownership status						
For-profit	-18.2 (-49.4 – 13.0)	0.734	5.3 (-27.4 – 16.9)	1.000	9.0 (-22.4 – 4.3)	0.443
Non-Profit	-25.6 (-57.4 – 6.2)	0.198	-8.9 (-32.0 – 14.1)	1.000	-4.6 (-18.4 – 9.2)	1.000
Government	-35.5 (-69.2 – -1.8)	0.033	-6.7 (-35.01 – 21.7)	1.000	-8.9 (-25.1 – 7.4)	0.892
Total beds						
Middle tercile	18.8 (10.2 – 27.4)	< 0.001	-1.4 (-8.8 – 5.9)	0.702	7.56 (1.9 – 13.2)	0.009
Highest tercile	29.4 (18.4 – 40.3)	< 0.001	-6.3 (-14.0 – 1.4)	0.108	12.0 (5.5 – 18.5)	< 0.001
Occupancy rate	-3.2 (-3.6 – -2.7)	< 0.001	-2.3 (-2.5 – -2.1)	< 0.001	-1.6 (-1.8 – -1.4)	< 0.001
Patient age	0.7 (0.1 – 1.3)	0.027	0.7 (0.2 – 1.3)	0.012	1.2 (0.8 – 1.7)	< 0.001
% female residents	0.02 (-0.4 – 0.4)	0.926	-0.1 (-0.4 – 0.3)	0.720	0.2 (-0.1 – 0.4)	0.211
% white residents						
Middle tercile	-22.5 (-32.9 – -12.0)	< 0.001	-1.7 (-8.1 – 4.6)	0.590	2.0 (-3.9 – 7.8)	0.506

© 2020 Braun RT et al. *JAMA Network Open.*

eTable 4. Association Between COVID-19 Resident Outcomes and Nursing Home Characteristics

	COVID confirmed cases, per 1,000 residents		COVID deaths, per 1,000 residents		All deaths, per 1,000 residents	
**Predictor**	**Risk estimate (95% CI)**	**P Value**	**Risk estimate (95% CI)**	**P Value**	**Risk estimate (95% CI)**	**P Value**
Highest tercile	-33.6 (-45.9 – -21.3)	< 0.001	2.5 (-6.4 – 11.3)	0.583	2.3 (-5.7 – 10.3)	0.568
% Medicare residents						
Middle tercile	-1.3 (-8.6 – 6.00)	0.729	5.5 (-0.8 – 11.8)	0.087	2.2 (-2.8 – 7.1)	0.395
Highest tercile	-7.3 (-17.0 – 2.3)	0.136	-4.6 (-11.2 – 2.1)	0.177	-3.3 (-8.7 – 2.3)	0.244
% Medicaid residents						
Middle tercile	7.6 (-1.2 – 16.4)	0.091	8.4 (1.9 – 14.8)	0.011	-1.5 (-7.3 – 4.3)	0.610
Highest tercile	7.8 (-2.7 – 18.3)	0.145	11.7 (4.5 – 18.9)	0.002	-2.5 (-9.0 – 3.9)	0.439
Mean ADL score	0.5 (-0.8 – 1.8)	0.443	0.7 (-0.5 – 2.0)	0.254	1.9 (1.1 – 2.7)	< 0.001
Multi-Facility Chain Membership	3.4 (-4.6 – 11.3)	0.404	0.4 (-5.4 – 6.1)	0.904	-1.6 (-6.3 – 3.2)	0.512
Rural	-15.6 (-26.1 – -5.0)	0.004	-19.5 (-34.7 – -4.3)	0.012	-15.9 (-23.1 – -8.6)	< 0.001

Abbreviations: CI, confidence interval; ADL, Activities of daily living
Results presented as difference in percentage points.
Bonferroni correction was used for multiple comparisons.
Standard errors were adjusted for clustering at the level of the Hospital Referral Region.

© 2020 Braun RT et al. *JAMA Network Open.*

eTable 5. Association Between PPE Supply Outcomes and Nursing Home Characteristics

	One-week supply of N95 masks		One-week supply of surgical masks		One-week supply of eye protection		One-week supply of gowns		One-week supply of gloves		One-week supply of hand sanitizer	
**Predictor**	**Estimate (95% CI)**	**P Value**	**Estimate (95% CI)**	**P Value**	**Estimate (95% CI)**	**P Value**	**Estimate (95% CI)**	**P Value**	**Estimate (95% CI)**	**P Value**	**Estimate (95% CI)**	**P Value**
Ownership status												
For-profit	9.1 (1.8 – 16.3)	0.006	1.6 (-6.2 – 3.1)	1.000	-0.1 (-4.7 – 4.5)	1.000	21.3 (11.8 – 30.8)	< 0.001	1.6 (-2.5 – 5.7)	1.000	1.0 (-3.2 – 5.3)	1.000
Non-Profit	13.0 (5.5 – 20.6)	<0.001	2.5 (-2.3 – 7.3)	1.000	3.7 (-1.2 – 8.6)	0.288	27.0 (17.7 – 36.2)	< 0.001	3.0 (-1.3 – 7.2)	0.386	2.7 (-1.8 – 7.3)	0.650
Government	14.8 (6.5 – 23.0)	< 0.001	2.4 (-3.0 – 7.8)	1.000	2.3 (-3.2 – 7.8)	1.000	25.7 (16.1 – 35.3)	< 0.001	3.3 (-1.0 – 7.7)	0.252	1.8 (-3.4 – 6.9)	1.000

© 2020 Braun RT et al. *JAMA Network Open.*

eTable 5. Association Between PPE Supply Outcomes and Nursing Home Characteristics

	**One-week supply of N95 masks**		**One-week supply of surgical masks**		**One-week supply of eye protection**		**One-week supply of gowns**		**One-week supply of gloves**		**One-week supply of hand sanitizer**	
**Predictor**	**Estimate (95% CI)**	**P Value**	**Estimate (95% CI)**	**P Value**	**Estimate (95% CI)**	**P Value**	**Estimate (95% CI)**	**P Value**	**Estimate (95% CI)**	**P Value**	**Estimate (95% CI)**	**P Value**
Total beds												
Middle tercile	-0.01 (-0.03 – 0.00)	0.135	-0.01 (-0.02 – 0.00)	0.085	-0.02 (-0.03 – 0.00)	0.010	-0.03 (-0.04 – -0.01)	0.005	-0.01 (-0.02 – 0.00)	0.105	-0.01 (-0.02 – 0.00)	0.019
Highest tercile	-0.02 (-0.04 – 0.00)	0.029	0.00 (-0.02 – 0.01)	0.595	-0.01 (-0.03 – 0.00)	0.042	-0.03 (-0.05 – -0.02)	< 0.001	-0.01 (-0.02 – 0.00)	0.227	-0.01 (-0.02 – 0.00)	0.182

© 2020 Braun RT et al. *JAMA Network Open.*

eTable 5. Association Between PPE Supply Outcomes and Nursing Home Characteristics

	**One-week supply of N95 masks**		**One-week supply of surgical masks**		**One-week supply of eye protection**		**One-week supply of gowns**		**One-week supply of gloves**		**One-week supply of hand sanitizer**	
**Predictor**	**Estimate (95% CI)**	**P Value**	**Estimate (95% CI)**	**P Value**	**Estimate (95% CI)**	**P Value**	**Estimate (95% CI)**	**P Value**	**Estimate (95% CI)**	**P Value**	**Estimate (95% CI)**	**P Value**
Occupancy rate	0.00 (0.00 – 0.00)	0.695	0.00 (0.00 – 0.00)	0.875	0.00 (0.00 – 0.00)	0.270	0.00 (0.00 – 0.00)	0.491	0.00 (0.00 – 0.00)	0.105	0.00 (0.00 – 0.00)	0.556
Patient age	0.00 (0.00 – 0.00)	0.750	0.00 (0.00 – 0.00)	0.306	0.00 (0.00 – 0.00)	0.016	0.00 (0.00 – 0.00)	0.068	0.00 (0.00 – 0.00)	0.921	0.00 (0.00 – 0.00)	0.789
% female residents	0.00 (0.00 – 0.00)	0.147	0.00 (0.00 – 0.00)	0.628	0.00 (0.00 – 0.00)	0.935	0.00 (0.00 – 0.00)	0.214	0.00 (0.00 – 0.00)	0.874	0.00 (0.00 – 0.00)	0.873

© 2020 Braun RT et al. *JAMA Network Open.*

eTable 5. Association Between PPE Supply Outcomes and Nursing Home Characteristics

	One-week supply of N95 masks		One-week supply of surgical masks		One-week supply of eye protection		One-week supply of gowns		One-week supply of gloves		One-week supply of hand sanitizer	
**Predictor**	**Estimate (95% CI)**	**P Value**	**Estimate (95% CI)**	**P Value**	**Estimate (95% CI)**	**P Value**	**Estimate (95% CI)**	**P Value**	**Estimate (95% CI)**	**P Value**	**Estimate (95% CI)**	**P Value**
% white residents												
Middle tercile	0.01 (-0.01 – 0.03)	0.507	0.01 (-0.01 – 0.02)	0.374	0.00 (-0.01 – 0.02)	0.894	-0.01 (-0.03 – 0.01)	0.206	0.01 (0.00 – 0.02)	0.260	0.00 (-0.02 – 0.01)	0.490
Highest tercile	0.00 (-0.03 – 0.03)	0.943	0.00 (-0.02 – 0.01)	0.624	-0.01 (-0.03 – 0.01)	0.343	-0.01 (-0.04 – 0.01)	0.369	0.00 (-0.01 – 0.02)	0.733	-0.01 (-0.03 – 0.01)	0.263
% Medicare residents												

© 2020 Braun RT et al. *JAMA Network Open.*

eTable 5. Association Between PPE Supply Outcomes and Nursing Home Characteristics

	One-week supply of N95 masks		One-week supply of surgical masks		One-week supply of eye protection		One-week supply of gowns		One-week supply of gloves		One-week supply of hand sanitizer	
**Predictor**	**Estimate (95% CI)**	**P Value**	**Estimate (95% CI)**	**P Value**	**Estimate (95% CI)**	**P Value**	**Estimate (95% CI)**	**P Value**	**Estimate (95% CI)**	**P Value**	**Estimate (95% CI)**	**P Value**
Middle tercile	0.01 (-0.01 – 0.02)	0.420	0.00 (-0.01 – 0.01)	0.735	0.00 (-0.01 – 0.01)	0.867	0.00 (-0.01 – 0.02)	0.727	0 (-0.01 – 0.00)	0.409	0.00 (-0.01 – 0.01)	0.555
Highest tercile	-0.01 (-0.03 – 0.01)	0.390	-0.02 (-0.03 – 0.00)	0.013	-0.01 (-0.02 – 0.01)	0.271	-0.01 (-0.03 – 0.01)	0.276	-0.01 (-0.02 – 0.00)	0.184	-0.01 (-0.02 – 0.00)	0.208
% Medicaid residents												

© 2020 Braun RT et al. *JAMA Network Open.*

eTable 5. Association Between PPE Supply Outcomes and Nursing Home Characteristics

	One-week supply of N95 masks		One-week supply of surgical masks		One-week supply of eye protection		One-week supply of gowns		One-week supply of gloves		One-week supply of hand sanitizer	
**Predictor**	**Estimate (95% CI)**	**P Value**	**Estimate (95% CI)**	**P Value**	**Estimate (95% CI)**	**P Value**	**Estimate (95% CI)**	**P Value**	**Estimate (95% CI)**	**P Value**	**Estimate (95% CI)**	**P Value**
Middle tercile	-0.01 (-0.03 – 0.00)	0.099	-0.01 (-0.02 – 0.01)	0.247	-0.01 (-0.02 – 0.00)	0.157	-0.01 (-0.03 – 0.01)	0.178	-0.01 (-0.02 – 0.00)	0.033	-0.01 (-0.02 – 0.00)	0.154
Highest tercile	-0.01 (-0.03 – 0.01)	0.492	0.00 (-0.02 – 0.02)	0.945	-0.01 (-0.02 – 0.01)	0.422	0.00 (-0.02 – 0.02)	0.785	-0.01 (-0.02 – 0.01)	0.219	-0.01 (-0.02 – 0.01)	0.409
Mean ADL score	0.00 (0.00 – 0.00)	0.574	0.00 (0.00 – 0.00)	0.705	0.00 (0.00 – 0.00)	0.523	0.00 (0.00 – 0.00)	0.355	0.00 (0.00 – 0.00)	0.871	0.00 (0.00 – 0.00)	0.625

© 2020 Braun RT et al. *JAMA Network Open.*

eTable 5. Association Between PPE Supply Outcomes and Nursing Home Characteristics

	One-week supply of N95 masks		One-week supply of surgical masks		One-week supply of eye protection		One-week supply of gowns		One-week supply of gloves		One-week supply of hand sanitizer	
**Predictor**	**Estimate (95% CI)**	**P Value**	**Estimate (95% CI)**	**P Value**	**Estimate (95% CI)**	**P Value**	**Estimate (95% CI)**	**P Value**	**Estimate (95% CI)**	**P Value**	**Estimate (95% CI)**	**P Value**
Multi-Facility Chain Membership	-0.01 (-0.03 – 0.00)	0.054	0.00 (-0.01 – 0.01)	0.522	-0.01 (-0.01 – 0.00)	0.325	-0.02 (-0.03 – 0.00)	0.019	0.00 (-0.01 – 0.01)	0.607	-0.01 (-0.01 – 0.00)	0.259
Rural	-0.01 (-0.04 – 0.01)	0.330	-0.01 (-0.03 – 0.01)	0.486	-0.01 (-0.03 – 0.01)	0.273	-0.02 (-0.05 – 0.01)	0.109	0.00 (-0.02 – 0.01)	0.558	-0.01 (-0.02 – 0.01)	0.509

Abbreviations: CI, confidence interval; PPE, personal protective equipment; ADL, Activities of daily living
Results presented as difference in percentage points.
Bonferroni correction was used for multiple comparisons.
Standard errors were adjusted for clustering at the level of the Hospital Referral Region.

© 2020 Braun RT et al. *JAMA Network Open.*

eTable 6. Association Between Staff Shortage Outcomes and Nursing Home Characteristics

	Shortage of nursing staff		Shortage of clinical staff		Shortage of aides		Shortage of other staff	
**Predictor**	**Estimate (95% CI)**	**P Value**	**Estimate (95% CI)**	**P Value**	**Estimate (95% CI)**	**P Value**	**Estimate (95% CI)**	**P Value**
Ownership status (%)								
For-profit	3.2 (-1.4 – 7.7)	0.405	0.3 (-1.5 – 2.1)	1.000	3.2 (-2.4 – 8.8)	0.788	1.0 (-2.6 – 4.7)	1.000
Non-Profit	1.0 (-4.0 – 6.0)	1.000	0.02 (-2.02 – 2.1)	1.000	1.0 (-5.4 – 7.3)	1.000	0.3 (-3.6 – 4.1)	1.000
Government	6.9 (0.02 – 13.9)	0.049	-0.2 (-3.0 – 2.7)	1.000	4.7 (-2.9 – 12.2)	0.601	2.5 (-2.6 – 7.7)	1.000
Total beds								
Middle tercile	0.02 (0.00 – 0.03)	0.070	0.01 (0.00 – 0.01)	0.135	0.03 (0.01 – 0.05)	0.010	0.01 (0.00 – 0.03)	0.082
Highest tercile	0.02 (-0.01 – 0.04)	0.159	0.00 (-0.01 – 0.01)	0.443	0.03 (0.01 – 0.06)	0.005	0.01 (-0.01 – 0.03)	0.202

© 2020 Braun RT et al. *JAMA Network Open.*

eTable 6. Association Between Staff Shortage Outcomes and Nursing Home Characteristics								
	**Shortage of nursing staff**		**Shortage of clinical staff**		**Shortage of aides**		**Shortage of other staff**	
**Predictor**	**Estimate (95% CI)**	**P Value**	**Estimate (95% CI)**	**P Value**	**Estimate (95% CI)**	**P Value**	**Estimate (95% CI)**	**P Value**
Occupancy rate	0.00 (0.00 – 0.00)	0.016	0.00 (0.00 – 0.00)	0.079	0.00 (0.00 – 0.00)	0.295	0.00 (0.00 – 0.00)	0.008
Patient age	0.00 (0.00 – 0.00)	0.167	0.00 (0.00 – 0.00)	0.848	0.00 (0.00 – 0.00)	0.775	0.00 (0.00 – 0.00)	0.572
% female residents	0.00 (0.00 – 0.00)	0.788	0.00 (0.00 – 0.00)	0.605	0.00 (0.00 – 0.00)	0.811	0.00 (0.00 – 0.00)	0.388
% white residents								
Middle tercile	0.00 (-0.02 – 0.02)	0.986	0.00 (-0.01 – 0.01)	0.948	0.00 (-0.02 – 0.03)	0.853	0 (-0.02 – 0.01)	0.687

© 2020 Braun RT et al. *JAMA Network Open.*

eTable 6. Association Between Staff Shortage Outcomes and Nursing Home Characteristics

	Shortage of nursing staff		Shortage of clinical staff		Shortage of aides		Shortage of other staff	
**Predictor**	**Estimate (95% CI)**	**P Value**	**Estimate (95% CI)**	**P Value**	**Estimate (95% CI)**	**P Value**	**Estimate (95% CI)**	**P Value**
Highest tercile	-0.01 (-0.04 – 0.01)	0.393	0.00 (-0.01 – 0.01)	0.854	-0.01 (-0.04 – 0.02)	0.487	-0.01 (-0.03 – 0.01)	0.433
% Medicare residents								
Middle tercile	-0.01 (-0.03 – 0.01)	0.426	-0.01 (-0.01 – 0.00)	0.134	-0.02 (-0.04 – 0.00)	0.045	-0.02 (-0.03 – 0.00)	0.015
Highest tercile	-0.02 (-0.04 – 0.00)	0.038	-0.01 (-0.02 – 0.00)	0.017	-0.03 (-0.05 – -0.01)	0.008	-0.02 (-0.04 – -0.01)	0.002
% Medicaid residents								
Middle tercile	0.01 (-0.01 – 0.02)	0.490	0.00 (-0.01 – 0.01)	0.826	0.01 (-0.01 – 0.03)	0.297	0.01 (-0.01 – 0.02)	0.358

© 2020 Braun RT et al. *JAMA Network Open.*

eTable 6. Association Between Staff Shortage Outcomes and Nursing Home Characteristics

	Shortage of nursing staff		Shortage of clinical staff		Shortage of aides		Shortage of other staff	
**Predictor**	**Estimate (95% CI)**	**P Value**	**Estimate (95% CI)**	**P Value**	**Estimate (95% CI)**	**P Value**	**Estimate (95% CI)**	**P Value**
Highest tercile	0.03 (0.01 – 0.05)	0.005	0.00 (-0.01 – 0.01)	0.894	0.04 (0.01 – 0.06)	0.004	0.02 (0.00 – 0.03)	0.034
Mean ADL score	0.00 (0.00 – 0.00)	0.698	0.00 (0.00 – 0.00)	0.73	0.00 (-0.01 – 0.00)	0.101	0.00 (0.00 – 0.00)	0.388
Multi-Facility Chain Membership	-0.02 (-0.03 – 0.00)	0.029	0.00 (-0.01 – 0.00)	0.446	-0.01 (-0.03 – 0.01)	0.187	-0.01 (-0.02 – 0.00)	0.164
Rural	0.00 (-0.03 – 0.04)	0.771	0.02 (0.00 – 0.03)	0.072	0.03 (0 – 0.06)	0.092	0.02 (0.00 – 0.05)	0.072

Abbreviations: CI, confidence interval; ADL, Activities of daily living
Results presented as difference in percentage points.
Bonferroni correction was used for multiple comparisons.
Standard errors were adjusted for clustering at the level of the Hospital Referral Region.

© 2020 Braun RT et al. *JAMA Network Open.*

eTable 7. Poisson Regression Estimates: Association Between COVID-19 Resident Outcomes and Ownership

	COVID confirmed cases, per 1,000 residents		COVID deaths, per 1,000 residents		All deaths, per 1,000 residents	
Predictor	Risk estimate (95% CI)	P Value	Risk estimate (95% CI)	P Value	Risk estimate (95% CI)	P Value
Nursing Home Characteristics						
Ownership status						
For-profit	-0.05 (-0.3 – 0.2)	1.000	-0.02 (-0.3 – 0.3)	1.000	-0.1 (-0.2 – 0.1)	0.635
Non-profit	-0.2 (-0.4 – 0.1)	0.465	-0.1 (-0.4 – 0.2)	1.000	-0.1 (-0.2 – 0.1)	1.000
Government	-0.4 (-0.8 – -0.04)	0.022	-0.1 (-0.5 – 0.4)	1.000	-0.1 (-0.3 – 0.1)	1.000
Total beds						
Middle tercile	0.4 (0.2 – 0.5)	< 0.001	0.02 (-0.1 – 0.1)	0.783	0.1 (0.1– 0.2)	0.001
Highest tercile	0.4 (0.3 – 0.55)	< 0.001	-0.1 (-0.2 – 0.1)	0.271	0.3 (0.1 – 0.2)	< 0.001
Occupancy rate	-0.03 (-0.04 – -0.03)	< 0.001	-0.03 (-0.03 – -0.03)	< 0.001	-0.02 (-0.02 – -0.02)	< 0.001
Mean ADL score	0.01 (-0.01 – 0.03)	0.309	0.01 (-0.01 – 0.03)	0.394	0.03 (0.02 – 0.04)	< 0.001
Multi-Facility Chain Membership	0.1 (-0.03 – 0.2)	0.210	0.01 (-0.1 – 0.1)	0.908	-0.01 (-0.1 – 0.1)	0.684

© 2020 Braun RT et al. *JAMA Network Open.*

eTable 7. Poisson Regression Estimates: Association Between COVID-19 Resident Outcomes and Ownership

	COVID confirmed cases, per 1,000 residents		COVID deaths, per 1,000 residents		All deaths, per 1,000 residents	
Predictor	Risk estimate (95% CI)	P Value	Risk estimate (95% CI)	P Value	Risk estimate (95% CI)	P Value
Rural	-0.7 (-1.1 – -0.3)	0.001	-0.4 (-0.9 – 0.01)	0.055	-0.3 (-0.4 – -0.1)	< 0.001
Patient Characteristics						
Patient age	0.01 (0.00 – 0.01)	0.169	0.02 (0.01 – 0.03)	< 0.001	0.02 (0.01 – 0.03)	< 0.001
% female residents	0.00 (-0.01 – 0.01)	0.991	0.00 (-0.01 – 0.00)	0.489	0.00 (0.00 – 0.00)	0.488
% white residents						
Middle tercile	-0.2 (-0.3 – -0.1)	< 0.001	-0.1 (-0.2 – 0.02)	0.131	0.00 (-0.1 – 0.1)	0.905
Highest tercile	-0.4 (-0.5 – -0.2)	< 0.001	-0.1 (-0.20– 0.1)	0.279	0.00 (-0.1 – 0.1)	0.939
% Medicare residents						
Middle tercile	0.01 (-0.1 – 0.1)	0.902	0.1 (0.01 – 0.2)	0.030	0.03 (-0.03 – 0.1)	0.265
Highest tercile	-0.1 (-0.2 – 0.1)	0.204	-0.1 (-0.2 – 0.04)	0.227	-0.04 (-0.1 – 0.03)	0.284
% Medicaid residents						

© 2020 Braun RT et al. *JAMA Network Open.*

eTable 7. Poisson Regression Estimates: Association Between COVID-19 Resident Outcomes and Ownership

	COVID confirmed cases, per 1,000 residents		COVID deaths, per 1,000 residents		All deaths, per 1,000 residents	
Predictor	Risk estimate (95% CI)	P Value	Risk estimate (95% CI)	P Value	Risk estimate (95% CI)	P Value
Middle tercile	0.1 (0.01 – 0.2)	0.034	0.2 (0.1 – 0.3)	0.002	0.00 (-0.1 – 0.1)	0.931
Highest tercile	0.1 (-0.02 – 0.2)	0.120	0.2 (0.1 – 0.3)	< 0.001	-0.02 (-0.1 – 0.1))	0.693

Abbreviations: CI, confidence interval; PPE, personal protective equipment; ADL, Activities of daily living
Bonferroni correction was used for multiple comparisons.
Standard errors were adjusted for clustering at the level of the Hospital Referral Region.

© 2020 Braun RT et al. *JAMA Network Open.*

eTable 8. Logistic Regression Estimates: Association Between PPE Supply/Staff Shortage Outcomes and Ownership

Outcome	For-profit Odds Ratio (95% CI)	P Value	Non-profit Odds Ratio (95% CI)	P Value	Government Odds Ratio (95% CI)	P Value
PPE Supply Measures						
One-week supply of N95 masks	2.0 (1.2 – 3.2)	0.001	2.9 (1.7 – 5.0)	< 0.001	3.7 (1.9 – 7.5)	< 0.001
One-week supply of surgical masks	0.77 (0.3– 1.8)	1.000	1.7 (0.7 – 4.2)	0.724	1.8 (0.6 – 5.4)	0.801
One-week supply of eye protection	1.0 (0.5 – 2.2)	1.000	2.2 (0.9 – 5.2)	0.093	1.6 (0.6 – 4.4)	1.000
One-week supply of gowns	3.8 (2.3 – 6.4)	< 0.001	7.3 (4.3 – 12.5)	< 0.001	6.5 (3.3 – 12.5)	<0.001
One-week supply of gloves	1.5 (0.6 – 3.3)	1.000	2.3 (0.9 – 5.7)	0.095	2.8 (0.9 – 8.2)	0.076
One-week supply of hand sanitizer	1.2 (0.6 – 2.8)	1.000	2.0 (0.8 – 5.0)	0.356	1.5 (0.4 – 5.3)	1.000
Staff Shortage Measures						
Shortage of nursing staff	1.4 (0.8 – 2.2)	0.528	1.1 (0.7 – 1.9)	1.000	1.8 (1.0 – 3.1)	0.056

© 2020 Braun RT et al. *JAMA Network Open.*

eTable 8. Logistic Regression Estimates: Association Between PPE Supply/Staff Shortage Outcomes and Ownership

Outcome	For-profit<br><br>Odds Ratio (95% CI)	P Value	Non-profit<br><br>Odds Ratio (95% CI)	P Value	Government<br><br>Odds Ratio (95% CI)	P Value
Shortage of clinical staff	1.2 (0.5 – 2.8)	1.000	1.0 (0.4 – 2.7)	1.000	0.97 (0.3 – 3.2)	1.000
Shortage of aides	1.3 (0.8 – 2.1)	0.965	1.1 (0.6 – 1.9)	1.000	1.4 (0.8 – 2.5)	0.631
Shortage of other staff	1.2 (0.7 – 2.0)	1.000	1.1 (0.6 – 1.9)	1.000	1.4 (0.7 – 2.6)	1.000

Abbreviations: CI, confidence interval; PPE, personal protective equipment
Bonferroni correction was used for multiple comparisons.
Standard errors were adjusted for clustering at the level of the Hospital Referral Region.

© 2020 Braun RT et al. *JAMA Network Open.*
